# *Candida auris* as an emerging fungal pathogen: Is climate change a perfect breeding ground for this fungal pathogen?

**DOI:** 10.22034/cmm.2025.345248.1612

**Published:** 2025-08-26

**Authors:** Raman Thakur

**Affiliations:** Department of Medical Laboratory Sciences, Lovely Professional University, Jalandhar-Delhi G.T. Road, Phagwara, Punjab, India

## Abstract

Over the past decade, there has been an increasing level of concern regarding *Candida auris*. This fungus was initially identified in the auditory canal of an elderly Japanese woman in a medical facility in Tokyo, Japan, in 2009. Over the course of the upcoming decade, the fungus emerged concurrently on four distinct continents, with each strain exhibiting sufficient genetic distinctiveness to dispel the notion of intercontinental disease transmission. With climate change, fungal infection rates have increased throughout the world. In the Indian subcontinent, the temperature has been rising over the years, which could be the leading reason for the increase in fungal infections. Hence, it is postulated that climate change can be the breeding ground for emerging fungal pathogens as they adapt themselves to high temperatures.

## Dear Editor

Iam writing to commend your journal for shedding light on the critical issue of *Candida auris* as an emerging fungal pathogen and to contribute further insights on the potential relationship between its proliferation and climate change. Over the past decade, there has been an increasing level of concern regarding *C. auris*. This fungus was initially identified in the auditory canal of an elderly Japanese woman in a medical facility in Tokyo in 2009 [ 1 ]. Over the course of the upcoming decade, the fungus emerged concurrently on four distinct continents, with each strain exhibiting sufficient genetic distinctiveness to dispel the notion of intercontinental disease transmission. 

With the climate change, fungal infection rates have increased throughout the world. In the Indian subcontinent, the temperature has been rising over the years [ 2 ], which could be the leading reason for the increase in fungal infections. Hence, it is postulated that climate change can be a breeding ground for emerging fungal pathogens as they adapt themselves to elevated temperatures. A study performed by Casadevall *et al*., suggests a potential correlation between the formation of *C. auris* and climate change, namely global temperature fluctuations, due to its observed thermotolerance. The aforementioned study concluded that *C. auris*, before being acknowledged as a human disease and becoming widespread in hospital settings, may have originally been an environmental fungus that potentially thrived as a plant saprophyte in specific ecosystems, such as wetlands [ 3 ]. The potential origin of its emergence could be associated with the impacts of global warming on wetland ecosystems. 

In another study conducted on the Andaman Islands, India, the researcher systematically gathered soil samples from a total of 48 distinct sites. These sites encompassed a diverse range of coastal ecosystems, including wetlands, rocky coasts, pristine sandy shorelines, tourist-populated beaches, tidal marshes, and mangrove swamps. The above-mentioned study has successfully identified and isolated two distinct strains of *C. auris*. One of the *Candida* species, found on a beach commonly visited by individuals, exhibited resistance to many drugs and showed a preference for higher temperatures that would typically be lethal to other *Candida* species. A distinct variant, exhibiting neither drug resistance nor heat adaptation, was discovered within a marshland region devoid of human habitation [ 4 ]. 

The "fungal infection-mammalian selection" hypothesis posits that the thermal barrier could potentially play a role in the spread of *C. auris*. This particular fungus is noteworthy as it may indicate a shift in the aforementioned equation, being the first known example of a fungus that has successfully adapted to a world experiencing climate change and consequently overcoming our thermal barrier [ 5 ]. Moreover, *C. auris* exhibits genetic plasticity, including chromosome variations, *ERG11* gene mutations, and efflux pump overexpression, enhancing antifungal resistance. Upregulation of HSP90 improves stress response and thermotolerance, aiding adaptation to environmental changes. Physiologically, its biofilm formation, salinity tolerance, and metabolic flexibility enable persistence and spread [ 6 , 7 ]. 

Therefore, this issue not only impacts healthcare but also highlights the need for a more robust and coordinated approach to infectious disease control. *C. auris* serves as a wake-up call to address these systemic shortcomings and strengthen healthcare infrastructures. Understanding the nexus between climate change and the emergence of fungal pathogens is imperative for devising proactive strategies. Enhanced surveillance of environmental reservoirs, investments in antifungal research, and interdisciplinary collaboration between mycologists, climate scientists, and public health experts are essential steps to mitigate the threat posed by *C. auris* and similar pathogens.

Emphasis of your journal on this critical topic is both timely and essential. I hope this letter contributes to the ongoing dialogue and underscores the urgency of integrating climate science into fungal pathogen research.
